# Dr. A. N. Denton

**Published:** 1901-03

**Authors:** 


### TEXAS MEDICAL JOURNAL.

AUSTIN, TEXAS.

A MONTHLY JOURNAL OF MEDICINE AND SURGERY.

EDITED AND PUBLISHED BY

F. E. DANIEL, M. D.

Published Monthly at Austin, Texas. Subscription price $1.00 a year in advance.

Eastern Representative: John Guy Monihan, St. Paul Building, 220 Broadway, New York City.

Official organ of the West Texas Medical Association, the Houston District Medical Association, the Austin District Medical Society, the Brazos Valley Medical Association, the Galveston County Medical Society, and several others.

### DR. A. N. DENTON.

DIED, in Austin, Texas, March 4th (inst.), after a lingering illness following apoplexy, Dr. Ashley Newton Denton, of Austin, in the 64th year of his age.

In the death of Dr. Denton, the medical profession loses one of its ablest and most learned members; the medical press a colleague, who, as editor of the *Texas Medical News* for several years, did some good work for the cause of medical and sanitary progress and reform. At the time of his death he was associate editor of the *Medical News.* He was a clear, concise and forceful writer, and contributed from time to time some valuable papers to the several medical societies of which he was a member,—the Texas State Medical Association and the Austin District Medical Society. He was president of the last named society in 1897. The State of Texas loses one of its most representative citizens, and the city of Austin is bereft of one of her most learned and esteemed physicians.

Dr. Denton was a man of high character and noble nature. In his whole professional life he lived up to the physicians' code, and ever stood for all that was high, noble, and best in professional, as in private life. He was pure in heart,—honorable in all his dealings,—his innate sense of right and justice,—his integrity and honesty,—the fearless expression of his convictions and defense of them, being strong characteristics. He was a man who never sought popularity, being reserved, modest and dignified; hence he had few intimates. Those who knew him best loved him most. Dr. Denton was superintendent of the State Lunatic Asylum in 1886-90,—during Governor Ireland's administration, and introduced many reforms, both in asylum management and the treatment of the unfortunate inmates. He made a special study of mental and nervous diseases, and had a wide reputation as an alienist and neurologist. He was a private in the Confederate army, and when discharged resumed the study of medicine—which he had begun before the war, and graduated from the Texas Medical College in 1866. He represented the Hays county district in the State Senate in 1874-6, and at one time took an active part in State politics; but for many years he devoted his whole time to his profession. He was greatly endeared to his patrons by his unselfish devotion to them in time of sickness, and many households will miss a staunch and sympathetic friend, who was ever ready to serve them. He leaves a wife and three sons and two daughters.

---

THE COMMITTEE reported adversely on the cumpulsory vaccination bill referred to elsewhere. The quarantine bill still hangs fire; tied up. The State Association of County Health Officers will meet in Austin March 21st, inst.

---

A "GENTLE REMINDER" that it takes money to pay the printer and to buy a straw hat and linen duster for the hot days that are coming, is sent out herewith to every fellow on the book who owes any subscription. We hope every fellow will *ante up*. Drafts are also drawn on a lot of fellows who have forgotten us three years and more. We hope they will ante up, too.

